# Supplementary material for: Correlation Fuzzy measure of multivariate time series for signature recognition
Source: PLoS One. 2024 Oct 7;19(10):e0309262. doi: 10.1371/journal.pone.0309262 (PMC11457994; doi:10.1371/journal.pone.0309262)
Supplement: Attachment — Submitted filename: Response to Reviewers.docx [file pone.0309262.s002.docx]

**Response to reviewers,**

**To Reviewer 1,**

**1. The symbols before and after the manuscript are quite confusing, which makes it difficult to understand.**

Response: Thanks for the meticulous work of the review on this manuscript. We check our script again and hope the revised one could be clarity.

As highlighted words, we have supplemented the intermediate process of the theoretical section. The format of formulas 4 and 5 has been re-edited. The symbol representation of covariance has been standardized.

As the general type of weighted dimension is

 (1)

The correlation distance is

(2)

Then, we have

(3)

As $\left\| \cdot\right\|$ is a norm of $\varphi$ and in a Banach space, it satisfies

(4)

As we also have, this deduces

(5)

Considering *F_1_* and *F_2_* have same distribution, the norm-equivalence theorem ensures

(6)

Where *M(t)* is decided by modulus of the finite segment in time series.

Let *X*, *Y* are two continuous random variances, *Cov(X,Y)* is their covariance, the correlation distance is calculated as follows

$\rho=\frac{Cov(X,Y)}{\sigma_{x}\sigma_{y}}$ (7)

, where $\sigma_{i}$ is the Standard Deviation.

We can use L_2_ norm to control the *x*-dimension measure under Correlation distance. We define a discrete Correlation distance of MTS with this note.

Let $T(n,x)$ is a discrete MTS, the discrete Correlation distance can be calculated as

$\rho(T_{i,\tau},T_{j,\tau})\text{=}\frac{Cov(\left\|T_{i,\tau}(n,t)\right\|_{2,x},\left\|T_{j,\tau}(n,t)\right\|_{2,x})}{\sigma_{i}\sigma_{j}}$ (8)

where $T_{m,\tau}$ means the *m-th* segment with length of $\tau$. denotes modulus of multi-dimension.

**2. The previous mathematical expression is purely theoretical and continuous, and there is no explanation or explanation on how to apply it to actual data later.**

Response: We have added a **diagram of the discretization method** and supplemented the intermediate process of discretization entropy as

**Fig. 1 the CFE scheme**

There is a general assumption here that the variance of a sequence is 1, but the actual sequence does not satisfy this property. Therefore, in actual calculations, we usually use the following formula, as

$S_{ij}^{\tau}=exp(-\ln2(\frac{\rho_{ij}^{\tau}\sigma_{i}\sigma_{j}}{r})^{m})=exp(-\ln2(\frac{Cov_{ij}^{\tau}}{r})^{m})$ (10)

**3 It is unclear how to apply it to unclear classification and how to combine it with other methods. Just providing some conclusions cannot determine the reliability of these conclusions.**

Response: Firstly, the signature sequences are calculated as CFE vectors; Then, the **Classification Toolbox of Matlab R2021b** is used in this experiment as in line 285-289

Generally, training signatures set 2 modes that using single samples or pairs samples. Here, we only discuss CFE on information of signature locus. It means that samples are two dimensions time series in single or in pairs. Then, CFE values are calculated as the inputs at τ={13, 14, 15, 16, 17, 18 19, 20}, corresponding to the result of **Section 3.** The Classification Toolbox of Matlab R2021b is used in this experiment.

**To Reviewer 2,**

**1. The manuscript is well written and the obtained results are satisfactory. To improve the quality of manuscript, I suggest to analysis the effect of time series length in accuracy of your proposed method. Please consider N=100, 200, 300 and 400 and compared the obtained results for some different examples.**

Response: Thanks for the meticulous work of the review on this manuscript.

Suggested by the respected reviewer, we have carried out simulations on length of N=100, 200, 300 and 400. At first, we consider the fact that the sequence points are randomly extracted from the signal, the 400 points are enough for our experiments. But now, we have more effective evidence. If you want to conduct more experiments, please run the code in the supporting materials. (reference to “readme.txt”)

As The sequence points are randomly extracted from the signal, and sequences of length 100-400 have similar performance in testing, we estimate CFE with N=400 time series and embedding dimension *τ* $\in${11, 12, …,20} in this report.

More details of simulation are as follows.

N=100

N=200

N=300

N=400

**Figure 1 error bar of entropy (FE, FPE and CFE from left to right)**

N=100

N=200

N=300

N=400

**Figure 2 std. of entropy (FE, FPE and CFE from left to right)**

N=100

N=200

N=300

N=400

**Figure 3 error and std. of sequence with noise (CFE)**
